# Recto-urethral fistula presenting as fever of unknown origin: a rare complication of prostatic abscess

**DOI:** 10.1590/S1677-5538.IBJU.2017.0468

**Published:** 2018

**Authors:** Sun Hwa Lee, Seong Jong Yun, Seokyong Ryu

**Affiliations:** 1 Department of Emergency Medicine, Sanggye Paik Hospital, Inje University College of Medicine, Seoul, Republic of Korea; 2 Department of Radiology, Kyung Hee University Hospital at Gangdong, Kyung Hee University School of Medicine, Seoul, Republic of Korea

## CASE DESCRIPTION

**Figure 1:**
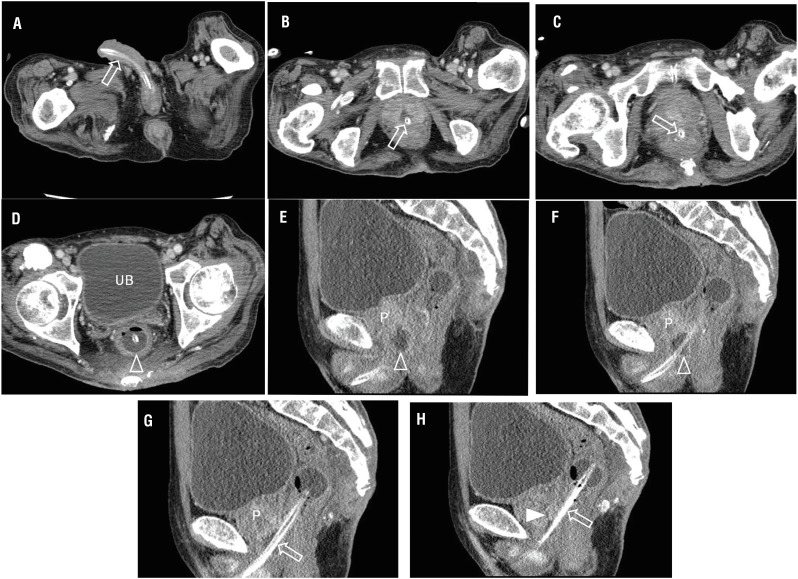
(A-D) Serial axial images of abdominal computed tomography shows malposition of the Foley catheter. It is located along the urethra (arrows, A) -prostate (arrow, B) -rectum (arrow, C), indicating presence of the recto-urethral fistula. The balloon (arrowhead, D) of the Foley catheter is located in the rectum, and not in the urinary bladder (UB). (E-H) Serial sagittal images of abdominal computed tomography reveals the penetration (arrows, G and H) of the prostate (P) with loculated fluid collection with air bubbles in the postero-inferior aspect of the prostate (open arrowhead, E and F), indicating a prostatic abscess. This abscess is bulging and abutting to the anterior wall of the rectum. Normal prostatic urethra is also seen (arrowhead, H). Additionally, there is soft tissue tumefaction involving the presacral space, indicating inflammation. However, bone window setting did not demonstrate bone involvement (no evidence of osteomyelitis).

A 76-year-old man was admitted in the emergency department complaining of fever of unknown origin for 1 month. His medical history was only significant for stroke, but there was no history of neoplasm, trauma, chemo-radiation, or other surgeries. He was admitted with a long-term Foley catheter in situ, which was inserted one year prior due to dysuria and changed regularly every 4-5 days. The present Foley catheter was inserted 4 days prior and the patient's urine color gradually changed and was dark green on presentation to hospital. Laboratory tests showed elevated white blood cell count (17.900/μL) and C-reactive protein (5.70mg/dL). In urine analysis, pyuria was seen. Abdominopelvic computed tomography (APCT) revealed there was no evidence of urinary tract infection or acute pyelonephritis. However, malposition of the Foley catheter was seen. It was located along the urethra-prostate-rectum (Figures 1A-1D). Also, a prostatic abscess between the prostatic urethra and rectum was bulging and abutting to the anterior wall of the rectum (Figures 1E-1H); thus, we diagnosed the recto-urethral fistula (RUF) caused by prostatic abscess. The patient was treated with intravenous antibiotics and percutaneous nephrostomy for urine diversion. Fecal diversion was not performed because fecaluria was not seen. The patient improved after three months of conservative treatment (pyelostomies) and he was discharged with Foley catheter reinsertion.

RUF is an abnormal connection between the rectum and urethra that is a rare complication of pelvic surgery, radiation, trauma, or infection/inflammation. The incidence of RUF has been on the rise due to an increase in the number of surgeries and pelvic irradiation performed for genitourinary neoplasm (1, 2). The early diagnosis of RUF using APCT in the emergency setting is important to not only confirm the diagnosis and initiate appropriate medical management, but also ensure pre-operative localization in patients that require surgery (3-5). In general, conservative management can be attempted by using urinary/fecal diversion for small (<2cm), simple RUF in non-irradiated patients who do not have sepsis. In contrast, large (≥2cm), complex RUF in irradiated patients may require surgical management (1, 2). Our case underscores physicians need to consider the possibility of RUF for early diagnosis and management in patients with risk factor for prostatic abscess or with history of recent low urinary tract procedure.
